# ﻿A pictorial key to the adult and larval nasal mites (Halarachnidae) of marine mammals

**DOI:** 10.3897/zookeys.1216.135359

**Published:** 2024-10-23

**Authors:** Morgan M. Shields, Tara Roth, Risa Pesapane

**Affiliations:** 1 Department of Veterinary Preventive Medicine, College of Veterinary Medicine, The Ohio State University, 1920 Coffey Rd., Columbus, OH 43210, USA; 2 San Mateo County Mosquito and Vector Control District, 1351 Rollins Rd., Burlingame, CA 94010, USA; 3 School of Environment and Natural Resources, College of Food, Agricultural, and Environmental Sciences, The Ohio State University, 2021 Coffey Rd., Columbus, OH 43210, USA

**Keywords:** Acari, dichotomous key, *Halarachnehalichoeri*, *Halarachnelaysanae*, *Halarachnemiroungae*, *Orthohalarachneattenuata*, *Orthohalarachnediminuata*

## Abstract

Mites in the family Halarachnidae are common endoparasites infesting the nasal tissues of a variety of marine mammals. These mites are easily transmissible and compromise the health of their hosts, especially in captive environments. While these mites are noted by marine mammal caretakers, they may easily be misidentified due to repeated revisions to halarachnid mite taxonomy and reclassification of misidentified specimens. Species identification currently requires multiple taxonomic keys, knowledge of revisions to species classifications through time, and training in acarology, which is impractical for marine mammal clinicians. Therefore, to summarize the known taxonomy and aid in future identification of halarachnid mites, we present a pictorial key composed of illustrations based on existing literature and images obtained by scanning electron microscopy (SEM) and high-resolution light microscopy (LM). Illustrations are organized into flow charts for the identification of both adult and larval stages. Dorsal shield silhouettes are also provided to facilitate the identification of adults. We hope that this key be used to simplify future taxonomic research, provide a standard for species identification, and aid in the diagnosis of halarachnid infestations in captive and rehabilitated marine mammal populations.

## ﻿Introduction

Five extant species of mites from two genera in the family Halarachnidae are known to infest a variety of marine mammals, including both captive and wild populations of pinnipeds and lutrinids (Furman and Dailey 1980; Pesapane et al. 2018). A sixth species, *Halarachneamericana* Banks, 1899, once infested the Caribbean monk seal (*Neomonachustropicalis* (Gray, 1850)) but is presumed extinct along with its host (Kenyon 1977; Furman and Dailey 1980). All species of halarachnids are parasitic and can be harmful to their hosts, impairing respiration by way of mucopurulent respiratory exudate, rhinitis, nasopharyngitis, bronchitis, and severe turbinate lysis (Dunlap and Piper 1976; Baker 1987; Alonso-Farré et al. 2012; Dent et al. 2019; Ebmer et al. 2022). Accurate identification of halarachnid mites is important for understanding host-parasite relationships, how to care for captive marine mammals, and the impact mite infestations may have on wild populations.

**Figure 1.**
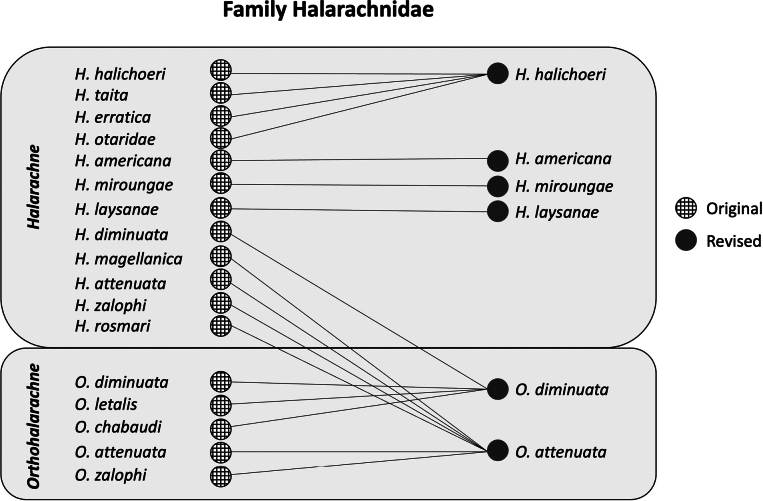
Graphic depiction of Halarachnidae systematics illustrating numerous taxonomic revisions from original species descriptions to revised current described species. Gridded and solid circles represent original and revised species names, respectively, and lines indicate synonymy.

The family Halarachnidae has undergone numerous taxonomic revisions (Fig. 1), which have invalidated some pre-existing keys and species descriptions (Domrow 1974; Furman and Dailey 1980), meaning that identification requires an in-depth literature review. Additionally, the limited number of well-preserved voucher specimens of some halarachnid species, similarity in morphology among halarachnid taxa, and varying degrees of host overlap by halarachnid mites have compounded the challenge of accurate identification. Although publications for halarachnid mite identification exist, they often focus on a single genus or describe a single species, meaning many documents are needed. Some of these publications are also difficult to obtain or describe extinct species such as *H.americana*. As a result, marine mammal clinicians find it challenging to accurately identify these mites, potentially missing valuable information on host specificity and behavior that could be used to identify sources of infestation and appropriate control methods. Parasite misidentification can stem from a lack of good quality specimens, training in acarology, and the use of outdated identification keys (Bush et al. 2021). Misidentification of parasites by veterinary pathologists or researchers can create error cascades leading to persistent misidentification of species in the literature and incorrect assumptions about parasite behavior and host preferences. While routine collaboration with acarologists is ideal, in practice this does not always happen due to logistical constraints or resource limitations. Therefore, pictorial guides that are accessible and understandable to clinicians and students can help avoid the consequences associated with misidentification. A composite morphological key is useful for both experts and non-experts in parasitology; the former because it ensures accurate taxonomy and future research, and the latter to recognize and accurately document trends in halarachnid infestations to improve marine mammal welfare.

We have produced a simple yet comprehensive pictorial guide to halarachnids based on published keys to increase accessibility and to aid in consistent identification of these mite species independent of their hosts. Our goal is for this key to be accessible to both parasitology experts and non-experts in order to further document and understand the impact of halarachnid infestations in both captive and free-ranging marine mammals.

## ﻿Methods

**Figure 2.**
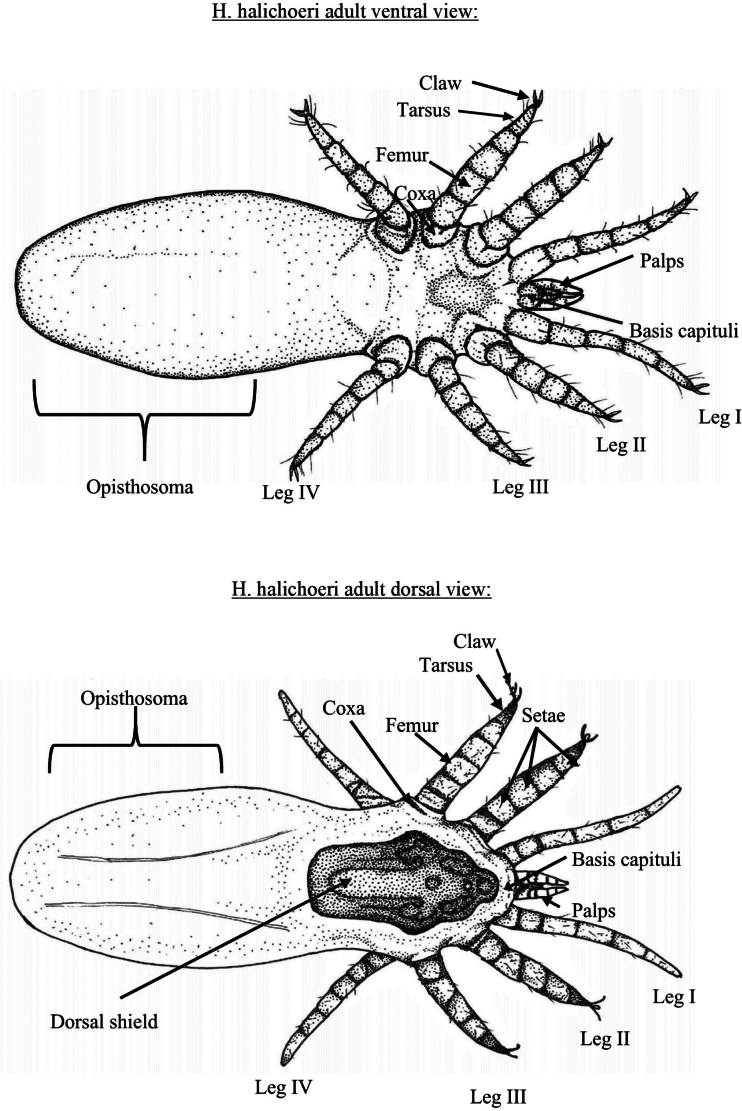
Key distinguishing morphological features of adult nasal mites (Halarachnidae).

**Figure 3.**
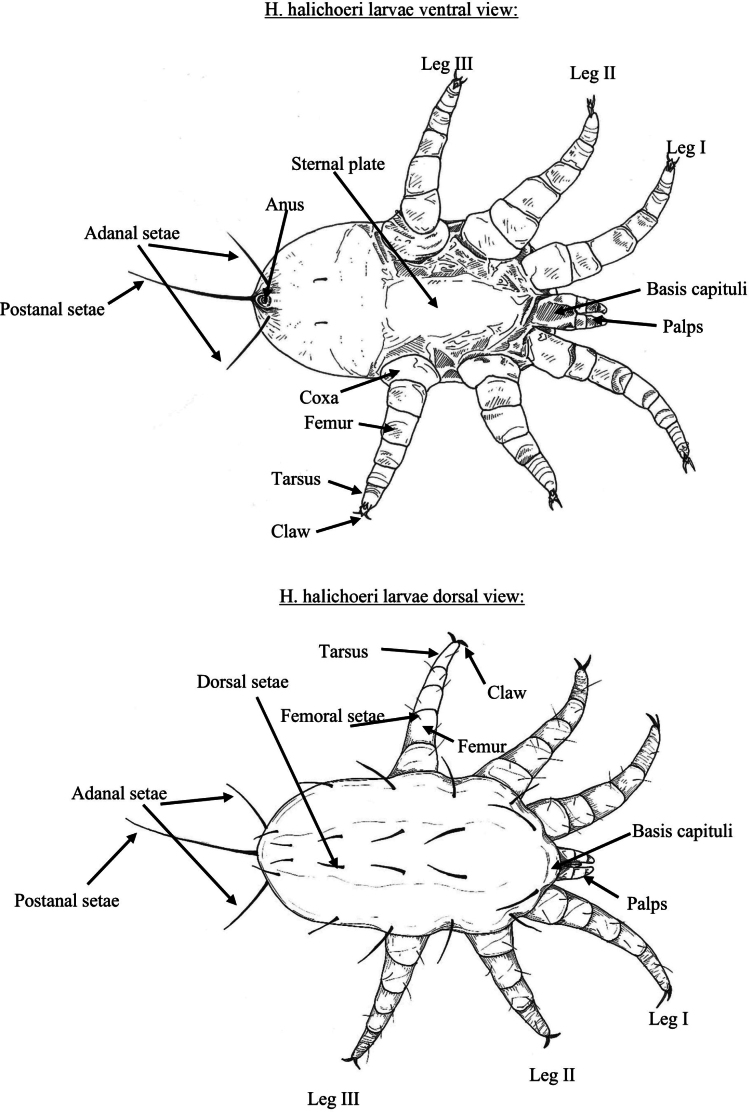
Key distinguishing morphological features of larval nasal mites (Halarachnidae).

A singular pictorial key for identifying larval and adult nasopulmonary mites from both *Halarachne* and *Orthohalarachne* was created using previously published morphologically distinguishing criteria and the most current taxonomic descriptions (Domrow 1962; Furman and Smith 1973; Furman and Dailey 1980; Alonso-Farré et al. 2012; Gastal et al. 2016; Rolbiecki et al. 2018; Ebmer et al. 2022). These criteria are clearly outlined in Figs 2, 3. The focus is on extant species, so *H.americana* is excluded from our key.

**Figure 4.**
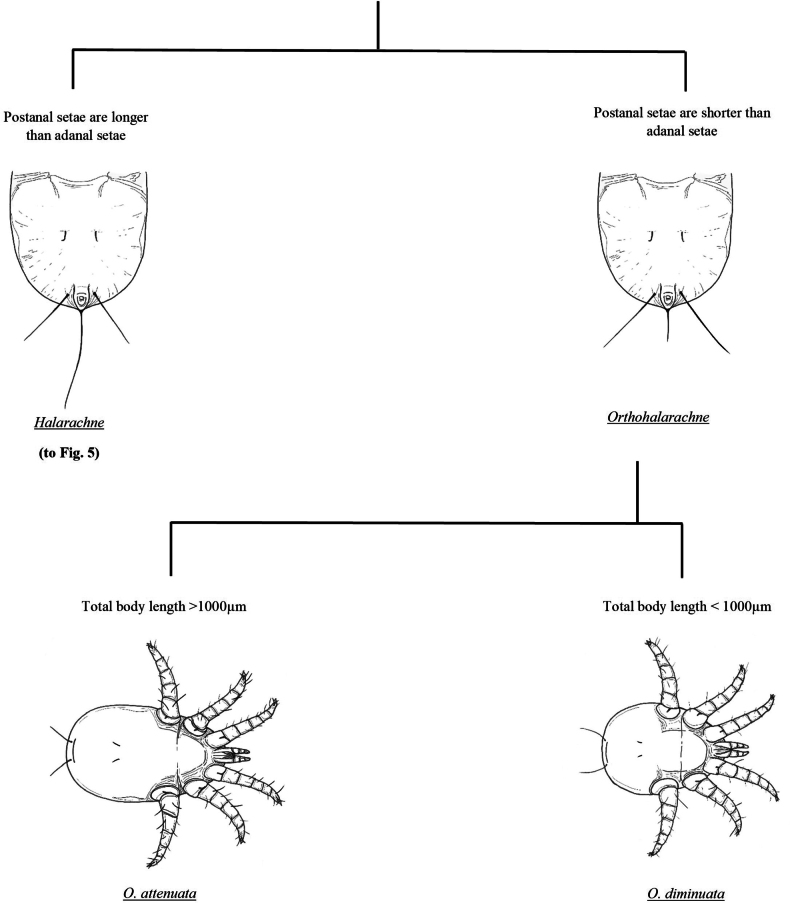
Pictorial key to the larval nasal mites (Halarachnidae) of marine mammals. Larvae are distinguishable from adult mites by the presence of six legs.

**Figure 5.**
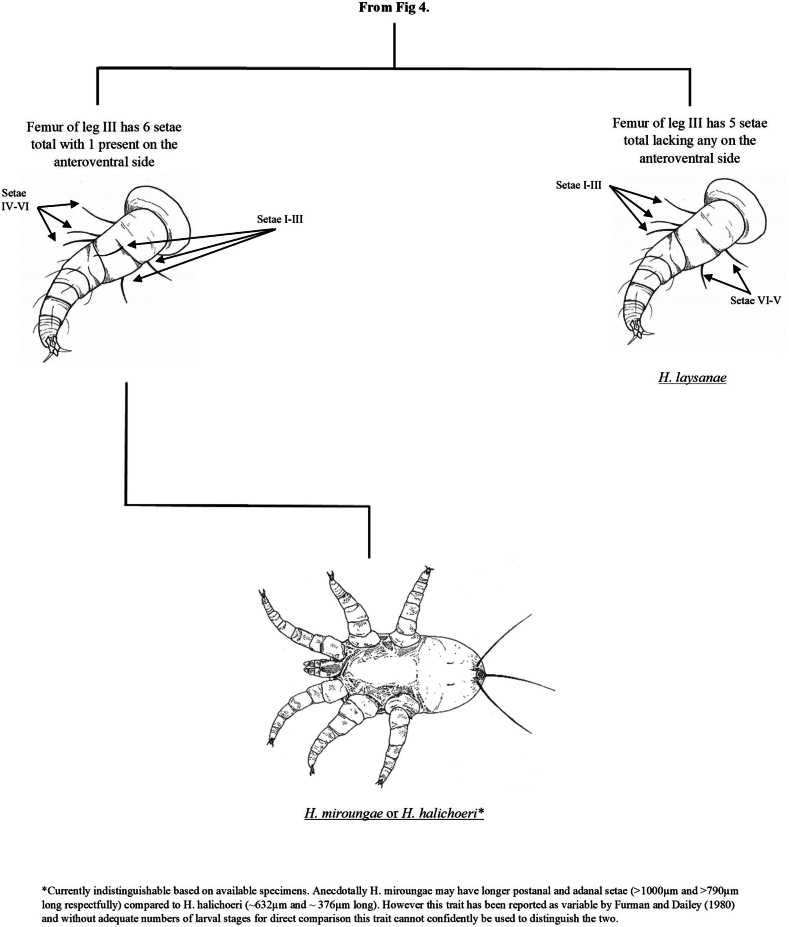
Pictorial key to the larval nasal mites (Halarachnidae) of marine mammals. Larvae are distinguishable from adult mites by the presence of six legs.

**Figure 6.**
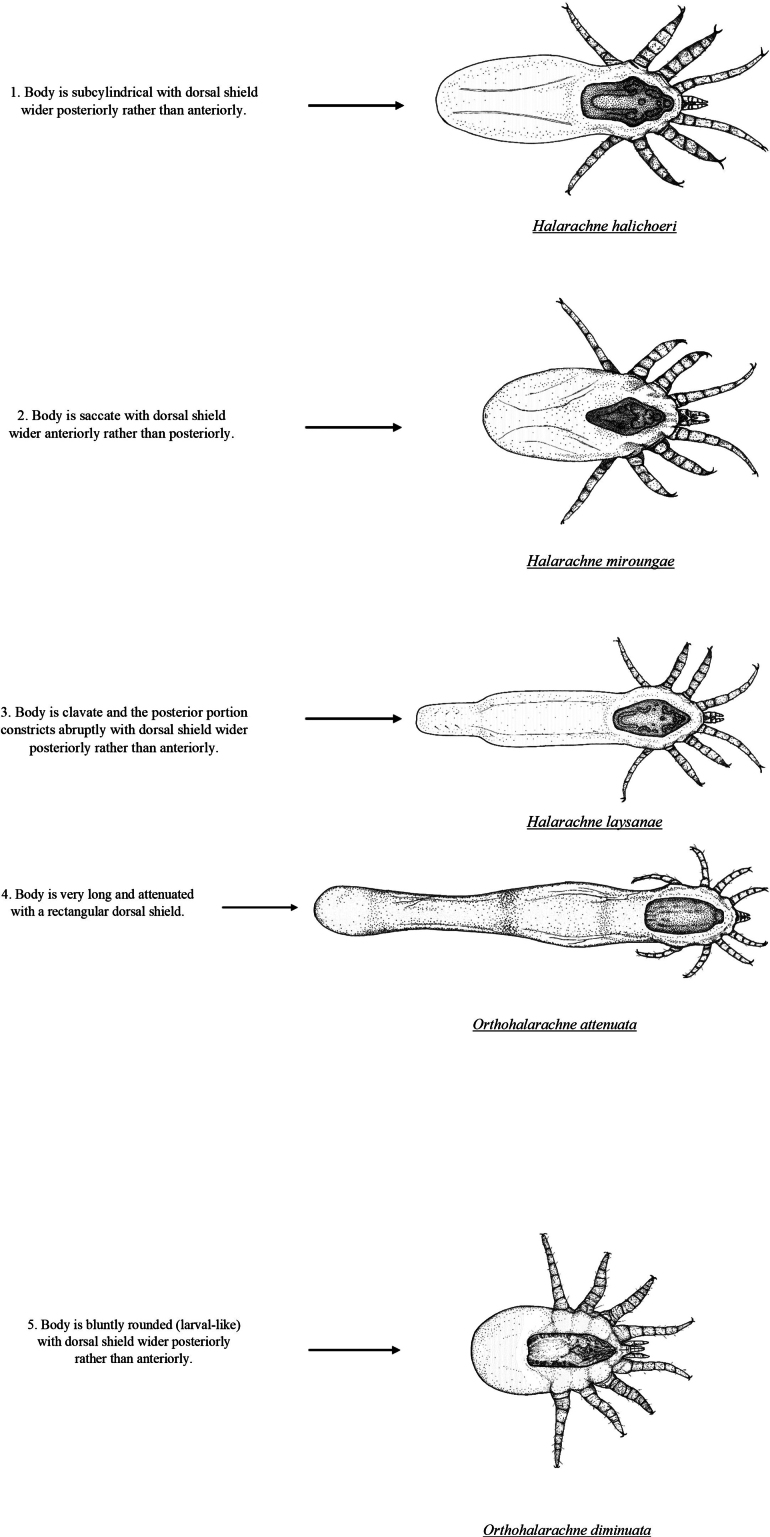
Pictorial key to the adult nasal mites (Halarachnidae) of marine mammals. Adult mites are distinguishable from the larval stage by the presence of eight legs.

**Figure 7.**
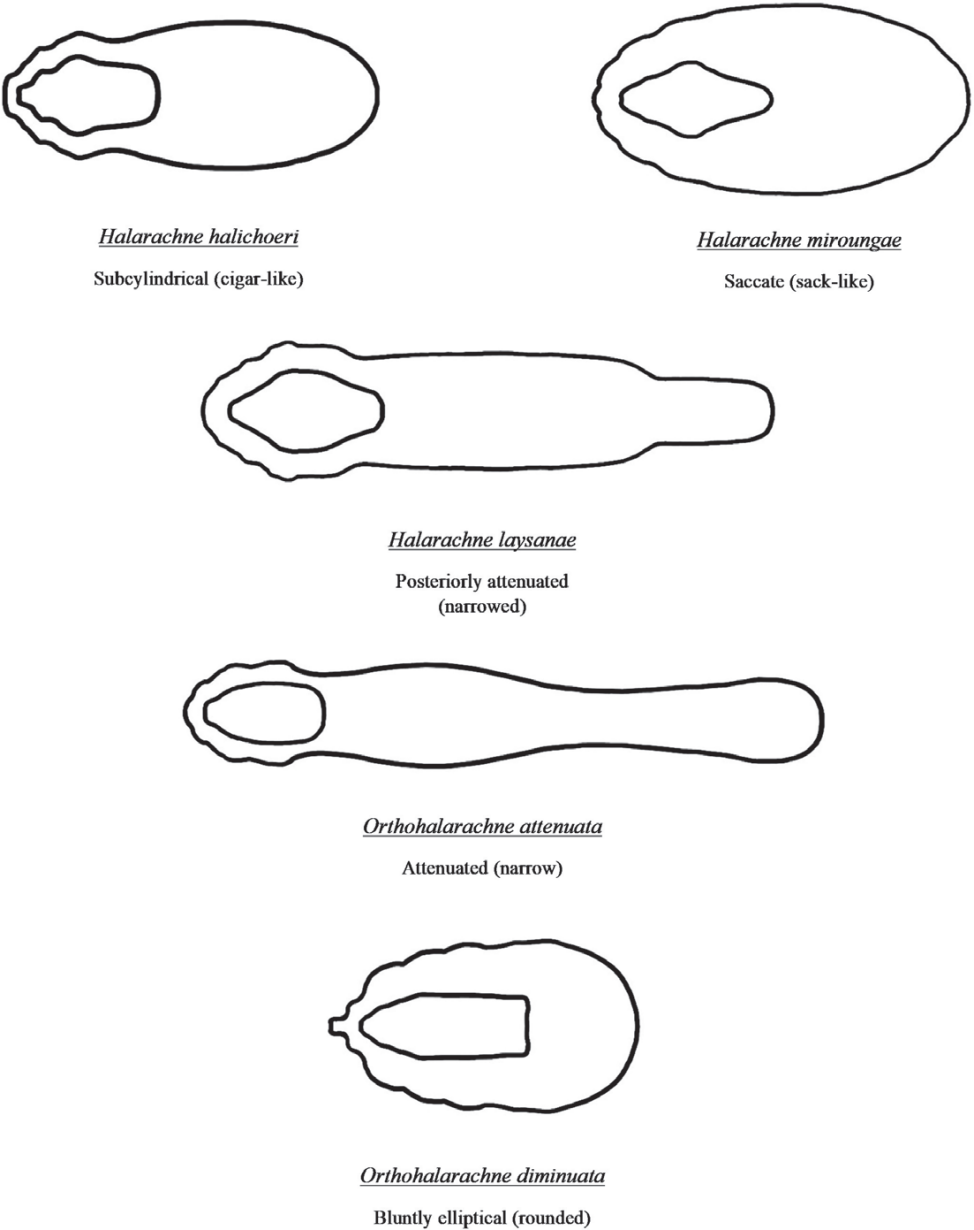
Body and dorsal shield outlines for simplified identification of adult halarachnids (anterior to the left, posterior to the right).

**Figure 8.**
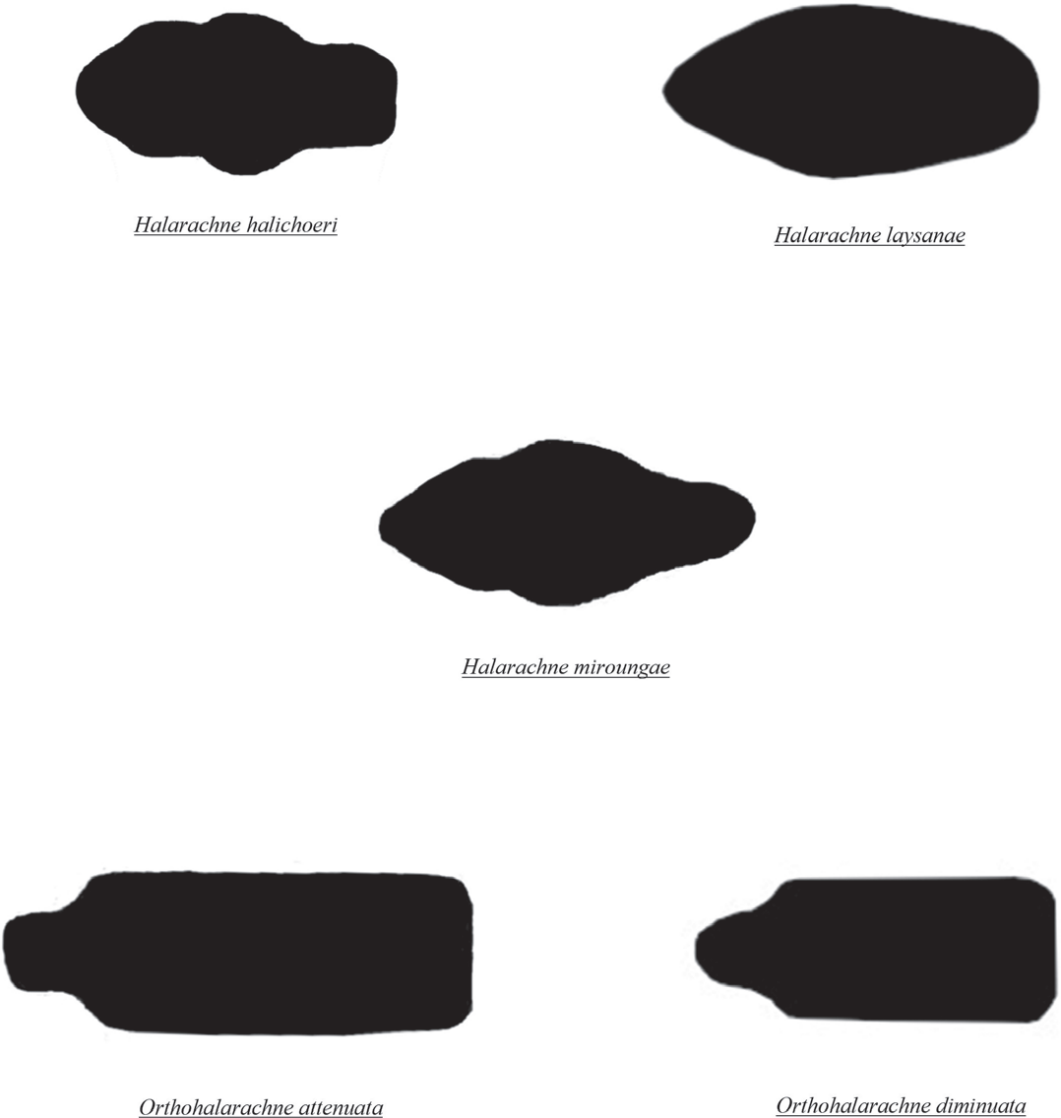
Dorsal shield shapes of adult halarachnids (anterior to the left, posterior to the right).

Fine-scale resolution of important defining morphologic characteristics were obtained from high-resolution light microscope (LM) images of 357 specimens of *H.halichoeri* Allman, 1847, *H.miroungae* Ferris, 1925, *O.attenuata* Banks, 1910, and adult *O.diminuata* Doetschman, 1944 in our archive using a Nikon SMZ25 stereomicroscope with DS-Ri2 camera (Nikon Inc., Melville, NY, USA). Additional images of *O.attenuata*, *O.diminuata*, and *H.miroungae* generated by both LM and scanning electron microscopy (SEM), and illustrations of *H.laysanae* Furman & Dailey, 1980 were gleaned from existing literature (Furman and Dailey 1980; Pesapane et al. 2018, 2021; Ebmer et al. 2022). Using these images, illustrations for the pictorial key were then hand drawn with pen and ink and organized into a flow chart figure for use in identifying both larval (Figs 4, 5) and adult (Fig. 6) halarachnids. This key also contains a figure depicting the body shape outline (Fig. 7) and dorsal shield shape of each adult species (Fig. 8). Dorsal shield and body morphology were outlined and excised from SEM and LM images using tools in the software NIS-Elements Basic Research (Nikon Inc., Melville, NY, USA), and images and descriptions from previous literature using GNU Image Manipulation Program (GIMP) v. 2.10.24 (https://www.gimp.org).

## ﻿Discussion

Adult halarachnid have distinct differences in body and dorsal shield shape making differentiation (particularly between genera) straightforward in this pictorial key. Adult *H.halichoeri* and *H.miroungae* share very similar morphology with two notable differences: *H.miroungae* opisthosoma (posterior end of the body) is more saccate (sack-like) than the subcylindrical (cigar-like) opisthosoma of *H.halichoeri* and the posterior portion of the dorsal shield of *H.halichoeri* is blunt and wider than the anterior portion, whereas in *H.miroungae* the posterior portion of the dorsal shield is pointed and narrower than the anterior portion. Adult *O.attenuata* are the most readily identified of the halarachnid mites because of their long opisthosoma that attenuates (becomes narrower) anteriorly to posteriorly. In contrast, the bluntly elliptical (rounded) opisthosoma of *O.diminuata* mimics the larval body form. Few images of well-preserved *O.diminuata* exist in the literature, making some features challenging to distinguish. For example, in Gastal et al. (2016) the shape of the dorsal shield was not well defined. The dorsal shield illustrations for *O.diminuata* included in this pictorial key are based on LM of our archival specimens, which agree with the shape depicted in Ebmer et al. (2022).

Identification of juvenile halarachnid mites is more challenging than adults. Larvae can be reliably identified to genus, but *H.miroungae* and *H.halichoeri* cannot be conclusively determined based on the current literature. While *H.miroungae* often has longer postanal and adanal setae compared to *H.halichoeri*, Furman and Dailey (1980) noted that this may be a variable characteristic, and we did not have larvae of *H.miroungae* in our archive for direct comparison. We have decided to forego using this feature in our key as a probable distinction between the two species as we note that a morphometric study of large sample sizes of both *Halarachne* species larvae is needed to confirm whether this characteristic is consistent enough for reliable species differentiation. Nymphal stages of halarachnid mites of either genus are rarely seen because these stages are teneral and of very short duration making them challenging for taxonomic identification by non-experts. For this reason, we have not included them in our pictorial key and recommend these specimens always be reviewed by an expert.

Halarachnid mites exhibit varying degrees of host specificity. Genus *Halarachne* infests primarily phocids and mustelids, while genus *Orthohalarchne* infests primarily otariids and odobenids (Rolbiecki et al. 2018; Pesapane et al. 2021). Within the genus *Halarachne*, *H.halichoeri* infests primarily harbor seals (*Phocavitulina* (Linnaeus, 1758)) and sea otters (*Enhydralutris* (Linnaeus, 1758)), while *H.miroungae* infests elephant seals (*Mirounga* spp. Gray, 1827), harbor seals, and sea otters (Fay and Furman 1982; Pesapane et al. 2018; Rolbiecki et al. 2018). The third species of this genus, *H.laysanae*, has only been found to infest Hawaiian monk seals (*Neomonachusschauinslandi* (Matschie, 1905)) (Fay and Furman 1982). Within genus *Orthohalarachne*, *O.attenuata* primarily infests northern fur seals (*Callorhinusursinus* (Linnaeus, 1758)), Cape fur seals (*Arctocephaluspusillus* (Schreber, 1775)), California sea lions (*Zalophuscalifornianus* (Lesson, 1828)), Guadalupe fur seals (*Arctocephalustownsendi* (Merriam, 1897)), and walrus (*Odobenusrosmarus* (Linnaeus, 1758)), while *O.diminuata* infests Stellar sea lions (*Eumetopiasjubata* (Schreber, 1776)), California sea lions, cape fur seals, and northern fur seals (Furman and Dailey 1980; Kim et al. 1980; Rolbiecki et al. 2018; Ebmer et al. 2022).

Although a relatively high degree of host specificity is a hallmark of the family Halarachnidae, some species may share hosts with other halarachnids. For example, *H.halichoeri* has been found to occasionally infest spotted seals (*Phocalargha* Pallas, 1811), hooded seals (*Cystophoracristata* (Erxleben, 1777)), California sea lions, and southern elephant seals (*Miroungaleonine* (Linnaeus, 1758)) (Rolbiecki et al. 2018). Accidental host spillover events have also been reported. *Halarachnehalichoeri* was found in a captive Gentoo penguin (*Pygoscelispapua* (Forster, 1781)) (Rolbiecki et al. 2018) and has been reported co-infesting a northern elephant seal (*Miroungaangustirostris* (Gill, 1866)) along with *H.miroungae* (Pesapane et al. 2021). *Orthohalarachneattenuata* and *O.diminuata* may co-infest cape fur seals, California sea lions, northern fur seals, and Stellar sea lions (Rolbiecki et al. 2018). Additionally, *O.attenuata* was recently identified as the first reported nasopulmonary mite infestation in the threatened Guadalupe fur seal, suggesting it may infest additional host species outside of those reported (Pesapane et al. 2021).

The host specificity of halarachnid mites may be a product of host behavior (such as dive depth) and anatomical adaptations. For example, *H.halichoeri* employs the use of a reinforced elastic tracheal trunk that can stay open at depths of 30–40 m (Pugh 1996a), whereas it is unlikely that *H.miroungae* employs the same methods, as the host it parasitizes often frequent depths of 300–400 m and the pressure at that depth would be too high for the tunica intima to hold the airway open (Pugh 1996a). This may explain why *H.halichoeri* seems to prefer shallow divers such as sea otters and harbor seals (Pesapane et al. 2018; Reckendorf et al. 2019). Generalist *Halarachne* larvae, unlike other acarids, do not seem to possess the olfactory chemoreceptors or sensilla required to distinguish between host species (Pugh 1996b). It is possible that such structures are not required as their hosts tend to form large rookeries, making it likely that larvae will come into contact primarily with conspecific hosts (Pugh 1996b).

The ecology of halarachnid mites and their effect on their associated host species can only be described through correct taxonomic identification. We have attempted to unite the myriad verbal descriptions, images, and revisionist publications of these genera into a single taxonomic key. This is an understudied group of organisms that may reveal interesting adaptations and behaviors to cope with changes in pressure, blood flow, temperature, or other environmental stresses. The accurate identification of these species is necessary to enable future behavioral and ecological research.

## ﻿Conclusion

The taxonomy of halarachnid mites has been subject to numerous revisions, and some publications on host associations have been controversial. Although some halarachnid species are readily distinguished, species within *Halarachne* are morphologically very similar, with only slight differences in the shape of certain attributes such as the dorsal shield or opisthosoma. Existing keys are numerous, frequently focus on a single genus or species, and contain relative comparisons like “more saccate” or “more subcylindrical”, which are difficult because they are subjective, and accuracy of species identification is best done via direct comparison. In this key, we compile all current taxonomic characteristics for species differentiation for both genera into a singular key with accompanying illustrations to aid in the easy and accurate identification of halarachnid mites in marine mammal hosts.

Misidentification of parasites has become a major issue (Bush et al. 2021), and this may lead to further errors in disease treatment and management, complicating animal recovery (Laga et al. 2021). This key will enable accurate halarachnid mite identification among experts and non-experts alike and can help alleviate some of the underlying drivers of misidentification. Improved accuracy and reporting of halarachnid mite infestations will also contribute to ongoing efforts to understand host–parasite relationships, managing mites in captive marine mammal populations, and evaluating the impact of mite infestations on wild populations.

## ﻿Additional information

### Author ORCIDs

Morgan M. Shields https://orcid.org/0009-0006-9268-413X

Tara Roth https://orcid.org/0000-0002-6046-0653

Risa Pesapane https://orcid.org/0000-0001-9318-9911

### Data availability

All of the data that support the findings of this study are available in the main text.
